# Increase of Tree Species Mingling enhances the resistance of *Pinus armandii* forests to *Dendroctonus armandi* infestation

**DOI:** 10.48130/forres-0025-0019

**Published:** 2025-09-16

**Authors:** Zhaolong Li, Xiaozi Zhou, Ziyan Zhang, Jie Fan, Yuanyong Dian

**Affiliations:** 1 College of Horticulture and Forestry Sciences, Huazhong Agricultural University, Wuhan 430070, Hubei, China; 2 Hubei Engineering Technology Research Centre for Forestry Information, Huazhong Agricultural University, Wuhan 30070, Hubei, China; 3 Key Laboratory of Urban Agriculture in Central China, Ministry of Agriculture, Wuhan 430070, Hubei, China

**Keywords:** *Dendroctonus armandi*, Topographic characteristics, Mingling, DBH, Infection probability

## Abstract

*Pinus armandii* Franch., a key native conifer in China, faces severe *Dendroctonus armandi* infestation, threatening forest ecosystems. Reducing infestation probability and enhancing resistance are essential for transforming *Pinus armandii* forests. This study investigates the correlation between stand structure, topographic factors, and the probability of *Pinus armandii* infestation by *Dendroctonus armandi*. Based on these correlations, it selects suitable mixed-species combinations of native tree species with low infestation probabilities that are adapted to regional characteristics. A random survey was conducted in 58 plots (6,021 trees) in Shennongjia. Logistic regression and analysis of variance (ANOVA) revealed: (1) Infestation rate increased with elevation, peaking at 84.38% above 2,000 m; between 1,500–2,100 m, probability rose 4.3% per 100 m elevation gain; (2) Steeper slopes (> 25°) reduced infestation (46.03%), with risk decreasing 1.9% per 1° slope increase (0°–40°); (3) Larger DBH (> 30 cm) trees had higher infestation (82.93%), increasing 4.5% per 1 cm DBH; (4) Higher species mingling (four neighboring non-Pinus trees) lowered infestation to 63.39%, reducing risk by 54.3% per mingling unit; (5) Healthy *Pinus armandii* were frequently neighbored by *Litsea pungens*, *Carpinus cordata*, *Phellodendron chinense*, and *Betula platyphylla*. Prioritizing slopes > 25° and elevations < 2,000 m for afforestation, mixed with *Litsea pungens*, *Carpinus cordata*, or *Betula platyphylla*, can mitigate infestation. These findings provide actionable strategies to enhance *Pinus armandii* forest resilience against *Dendroctonus armandi* threats.

## Introduction

*Pinus armandii* Franch., a species of evergreen tree in the subgenus *Strobus* of the genus *Pinus* (Pinaceae), is a native plant of China, widely distributed in southern Shaanxi (Qinling mountains), western Hubei, Sichuan, and other regions. It is not only an excellent timber and landscape tree species but also plays a crucial role in ecological conservation. However, *P. armandii* forests are highly susceptible to infestation by *Dendroctonus armandi*. *D. armandi*, an insect of the order Coleoptera, family Ipidae, and genus Dendroctonus, is a highly specialized pest that poses a severe threat to *P. armandii*. *D. armandi* primarily feeds on the phloem of its host and, in conjunction with blue-stain fungi, can cause the rapid death of *P. armandii* within just 60 d^[1]^. As a typical pioneer pest, *D. armandi* frequently triggers severe localized forest infestations^[2]^. Notably, the Shennongjia forest region, one of the core distribution areas of *P. armandii* outside the Qinling mountains, is renowned for its rich biodiversity and unique ecological value^[3]^. Its distinctive geographic environment and climatic conditions provide an ideal habitat for *P. armandii* but also facilitate the spread of *D. armandi*. Studies have shown that *D. armandi* exhibits strong ecological adaptability in this region, and its infestation has become increasingly frequent, with the affected area expanding over recent years^[4]^. As of 2018, approximately 6,700 ha of *P. armandii* forests in the Shennongjia region had been damaged by *D. armandi*^[5]^, resulting in severe degradation of forest resources and ecological environments^[5]^. Currently, most *P. armandii* forests in the region are artificially planted and widely distributed. Identifying the factors influencing the probability of *P. armandii* infestation and selecting tree species combinations with lower infestation risks to improve stand resistance through forest restructuring are key challenges in the conservation and restoration of *P. armandii* forests^[6]^. A fundamental scientific question to address is understanding the factors that make *P. armandii* forests highly vulnerable to *D. armandi* infestation. However, existing studies have primarily focused on the spatial distribution and biological characteristics of *D. armandi*, as well as the correlations between topography, stand structure, and tree mortality. Therefore, investigating the factors affecting the resistance of *P. armandii* to *D. armandi*, analyzing the combined effects of topographic and stand characteristics on pest outbreaks, and exploring afforestation strategies to mitigate infestation risks will provide a theoretical foundation for effective pest management and control. Moreover, these insights will contribute to the sustainable management and conservation of *P. armandii* forests.

Topographic factors, such as elevation, slope, and aspect, significantly influence the distribution of forest pests and the extent of infestations by altering ecological factors such as temperature, humidity, light intensity, soil conditions, and moisture distribution^[7,8]^. However, research findings indicate that the effects of topography vary across different regions. High-altitude areas typically experience lower temperatures and greater temperature fluctuations, which may impact pest survival rates and reproductive cycles^[9]^. Meanwhile, slope and aspect regulate solar radiation and hydrothermal resources, thereby influencing the spread of *D. armandi*. For instance, gentle slopes tend to accumulate more moisture, which may be conducive to host tree growth but could also create more favorable conditions for pest activity. In contrast, steeper slopes may restrict pest movement but could also affect the survival of both host trees and their natural enemies. Additionally, topography indirectly influences pest outbreaks by regulating the dynamic balance between host plants and predatory insects. Existing studies suggest that complex topographic structures alter the distribution patterns of matter and energy within forests, thereby affecting the spatial distribution of forest pest infestations^[10]^. For example, some studies have found that changes in aspect significantly impact insect reproductive success and dispersal patterns by modifying light and thermal conditions^[11]^. Therefore, in different regions, the impact of topography on the probability of *P. armandii* infestation by *D. armandi* varies, and in some cases, opposing trends may be observed. Thus, it is crucial to clarify the influence of topographic factors specific to the Shennongjia forest region.

The composition and structural characteristics of forest stands also play a crucial role in pest infestations. However, studies on the relationship between tree species diversity and forest pest diversity have yielded mixed results. Similarly, while mixed-species forests are often suggested as a means to reduce pest risks, there is no clear consensus on the most effective tree species combinations for different regions. Some studies have compared the impact of insect infestations in pure forests vs mixed forests with varying numbers of tree species. The results indicate a negative correlation between tree species composition and pest diversity^[10,12,13]^. This is primarily because non-host plants in mixed forests dilute host resources for specialized pests while also enhancing overall forest ecological stability.

However, other studies have found that tree species diversity and pest abundance do not always exhibit a strictly negative correlation but rather follow a unimodal relationship^[12]^. Previous research has highlighted that *D. armandi* is highly sensitive to changes in host density and distribution patterns. In stands dominated by a single tree species, pests tend to aggregate, leading to a higher risk of infestation^[14]^. As a result, to reduce the probability of pest outbreaks, converting pure *P. armandii* forests into mixed forests has become a widely adopted strategy^[15]^. However, there is currently no definitive research identifying the optimal tree species combinations for *P. armandii* mixed forests to minimize *D. armandi* infestation risk. Moreover, due to variations in tree species adaptability across different regions, the most suitable species for mixing with *P. armandii* may differ depending on the specific regional conditions.

Existing studies have shown that *D. armandi* exhibits a preference for infesting mature *P. armandii* trees over 30 years old across multiple regions^[16]^. For example, research conducted in the Qinling region of Shaanxi has demonstrated that *D. armandi* exhibits significant selectivity based on the age structure of individual trees. However, although tree age has been extensively studied, there is still a lack of systematic research on the combined effects of other individual tree characteristics, topographic factors, and stand structure on the ability of *P. armandii* to resist pest infestations.

Therefore, the objective of this study is to investigate the correlation between topographic factors (such as elevation, slope, and aspect), individual tree characteristics, and the spatial structure of *P. armandii*, and the probability of *P. armandii* being infested by *D. armandi* in the Shennongjia region. This research aims to provide a basis for the restoration of infested *P. armandii* forests and the selection of suitable sites for afforestation. Additionally, by analyzing the most frequently occurring neighboring tree species around healthy *P. armandii* under different topographic conditions, this study seeks to establish a theoretical foundation for pest control through stand structure adjustments.

## Materials and methods

### Study area

**Figure 1:**
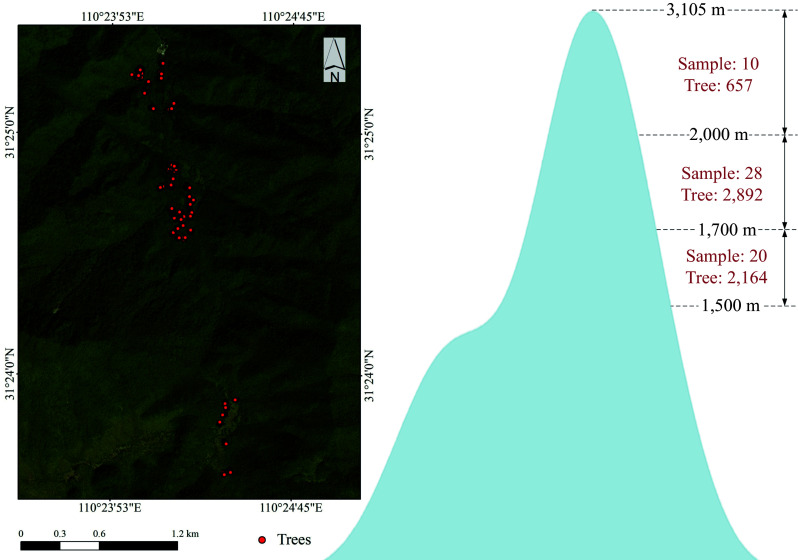
Study area showing the locations of individual tree surveys and the number of individual trees surveyed at different elevation gradients.

The Shennongjia forest region (110°23'7"−110°37'37" E, 31°18'44"−31°28'4" N) is located in northwestern Hubei Province, China. The Shennongjia mountain range within this region has a maximum elevation of 3,105 m and a minimum elevation of 398 m, resulting in significant topographic variation, which provides natural conditions for ecological studies under different terrain conditions. Shennongjia is renowned for its rich biodiversity and the presence of numerous nationally protected wild plant and animal species. It serves as a transitional zone between the northern subtropical monsoon climate and the temperate climate, exhibiting distinct transitional climatic characteristics. The region has an annual average temperature of 11.0–12.2 °C, and an annual precipitation of 800–1,200 mm, with synchronized rainfall and heat, creating favorable conditions for various forest vegetation types. As a dominant tree species in the alpine forest areas of Shennongjia, *P. armandii* is widely distributed at high elevations and constitutes a crucial component of the regional ecosystem. The sample plots in this study were primarily located in the high-altitude areas of Shennongjia National Forest Park, ensuring representativeness (Fig. 1).

### Individual tree survey

This study conducted sample plot surveys along an elevation gradient during 2021 and 2022. Given the complex forest environment in the study area, the diversity of topographic factors in the survey data and the accessibility of plots were both accounted for. Consequently, a random sampling approach was adopted along the elevation gradient. A total of 6,021 individual trees with a diameter at breast height (DBH) greater than 5 cm were recorded across 58 randomly selected plots, each measuring 20 m × 20 m. Among them, 2,048 were *P. armandii*, of which 1,392 were infested by *D. armandi*, while 656 remained non-infested. The number of trees surveyed at different elevations is shown in Fig. 1. Using the five-tree method, the location, species, DBH, tree height, crown width, canopy density, and topographic characteristics for each *P. armandii* and its four nearest neighbors were recorded. Regarding slope aspect, since slope direction affects solar radiation, which in turn alters the energy flux and moisture exchange between the atmosphere and the surface, creating local microclimates that influence vegetation growth^[17]^, the study area is located in the Northern Hemisphere. In the survey, based on actual solar radiation duration and intensity variations, and referring to other studies on slope aspect classification, a slope aspect with azimuth angles of 67°−247° was categorized as the sunny slope, while azimuth angles of 0°−67° and 247°−360° were classified as the shady slope^[17,18]^.

Since *D. armandi* larvae and adults primarily parasitize the inner phloem and outer xylem of *P. armandii*, feeding and reproducing extensively within these layers, infested trees exhibit distinct symptoms. At the entry holes, *P. armandii* exudes resin, which combines with frass and excrement expelled from the galleries, forming a funnel-shaped deposit. Meanwhile, the tree crown gradually turns yellow, and within 1–3 years after infestation, the tree dies^[19]^. Based on these characteristics, the infection status of each *P. armandii* was identified and recorded.

This study analyzes the association between various factors, and the probability of *P. armandii* infestation based on observational data. However, due to the nature of the data, the factors strongly correlated with the infestation probability do not necessarily imply direct causality for changes in tree infestation risk.

### Horizontal structural diversity

**Figure 2:**
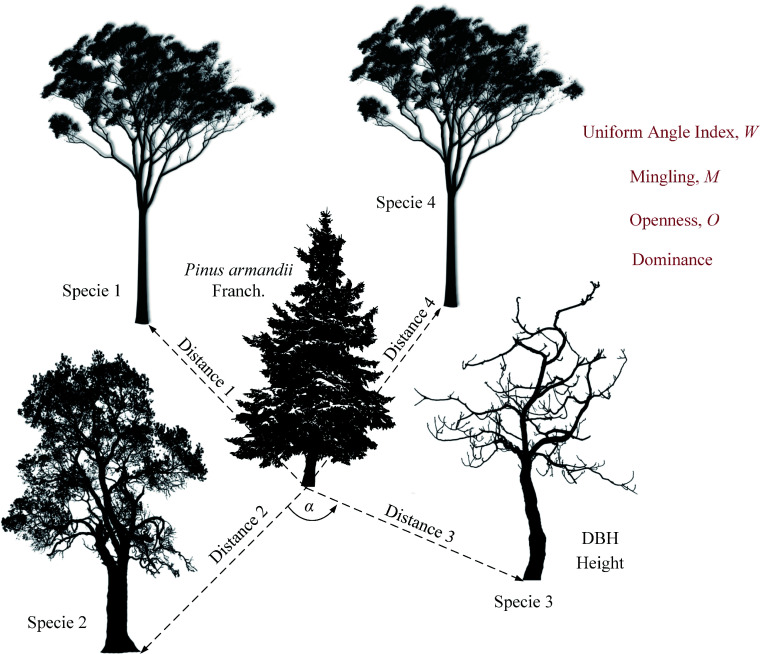
Horizontal structural indices for the four nearest neighboring trees around the focal *P. armandii* tree. Here, *α* denotes the parameter for calculating the uniform angle index, and 'Distance' represents the distance from a neighboring tree to the focal *P. armandii*. The trees in the diagram are for illustration purposes only and do not represent specific species.

This study employed multiple structural indices to quantify the diversity characteristics of tree horizontal structures. The indices included Uniform Angle Index, Mingling, Height Dominance, DBH Dominance, and Openness, all of which range from 0 to 1^[13,20−22]^. Figure 2 presents a schematic diagram of the calculation methods. The following sections provide detailed definitions and explanations of each index.

The Uniform Angle Index (*W*) is an indicator used to quantify the spatial distribution pattern of trees at the horizontal level. This index measures the proportion of neighboring trees with an angle (*α*) smaller than a standard angle (*α*_0_), which is typically set at 72° (i.e., 360°/5). The calculation is given in Eq. (1), where *n*_*α*_ < *α*_0_ represents the number of neighboring trees with an angle smaller than the standard angle, and *N* is the total number of nearest neighboring trees (four trees). When *W* approaches 1, it indicates a clustered tree distribution, whereas when *W* approaches 0, it suggests a more uniform horizontal distribution. The values of *W* are fixed at five discrete levels: 0, 0.25, 0.5, 0.75, and 1.

1 $ W=\dfrac{{n}_{\alpha\, <\, {\alpha }_{0}}}{N} $

The Mingling Index (*M*) is an indicator that reflects the degree of spatial species segregation. Specifically, it represents the proportion of the four nearest neighboring trees that belong to a different species from the reference tree. The calculation is given in Eq. (2), where *n*_*diff species*_ represents the number of neighboring trees with a different species from the reference tree, and *N* is the total number of nearest neighbors (four trees). A higher *M* value indicates a higher degree of tree species mixture within the stand. The possible values for *M* are fixed at 0, 0.25, 0.5, 0.75, and 1.

2 $ M=\dfrac{{n}_{dif f\;species}}{N} $

The Dominance Index (*D*) quantifies the size differences between the reference tree and its neighboring trees, including Height Dominance and DBH Dominance, which describe the relative advantage of the reference tree in terms of height and DBH, respectively. The calculation is given by Eq. (3), where *n*_*larger neighbors*_ represents the number of neighboring trees that are larger than the reference tree in terms of height (for Height Dominance) or DBH (for DBH Dominance), and *N* is the total number of nearest neighbors (four trees). A *D* value closer to 1 indicates that the neighboring trees have a greater size advantage over the reference tree. The possible values for *D* are 0, 0.25, 0.5, 0.75, and 1.

3 $ D=\dfrac{{n}_{larger\;neighbors}}{N} $

The Openness Index (*O*) measures the degree of spatial openness between the reference tree and its neighboring trees by assessing the ratio of the horizontal distance to the neighboring trees relative to their height. The calculation is given by Eq. (4), where *d*_*i*_ represents the horizontal distance between the reference tree and the i-th neighboring tree, and *h*_*i*_ represents the height of the i-th neighboring tree. A higher *O* value indicates greater spatial openness between trees.

4 $ O=\dfrac{{\displaystyle\sum }_{i=1}^{N}\dfrac{{d}_{i}}{{h}_{i}}}{N} $

### Logit model and variance analysis with ICC calculation

To preliminarily assess the independence among trees, this study first calculated the intraclass correlation coefficient (ICC[1]) to verify whether there is significant similarity among trees within the same plot. ICC[1] quantifies the variation between plots and evaluates the contribution of random effects to the probability of *P. armandii* infestation. A low ICC[1] indicates that trees are largely independent, thus meeting the independence assumption required by logistic regression models for the data^[23]^. The calculation method is shown in Eq. (5):

5 $ {\mathrm{ICC[1]}}=\dfrac{{\sigma }_{d}^{2}}{{\sigma }_{d}^{2}+{\sigma }_{r}^{2}} $

In Eq. 5, $ {\sigma }_{d}^{2} $ and $ {\sigma }_{r}^{2} $ are the estimates of the variances for the random intercept and random residual effects^[24]^.

To preliminarily assess the effects of individual tree characteristics, horizontal structural attributes, and topographic features on the probability of *P. armandii* infestation, this study first conducted a one-way analysis of variance (ANOVA) on key variables. Specifically, an independent two-sample t-test was used to examine whether there were statistically significant differences between infested and non-infested trees, thereby identifying potential variables influencing infestation probability.

Building upon the results of the ANOVA, this study further employed a Logistic Regression (Logit) model to conduct a multifactor analysis of infestation probability. The advantage of the Logit model lies in its ability to simultaneously account for multiple variables' combined effects on infestation risk while evaluating the independent effect of each variable. Using infestation status (1 for infested, 0 for non-infested) as the dependent variable, multiple independent variables were introduced. The model quantifies the impact of each independent variable on the dependent variable using log-odds transformation, a commonly used method for binary data analysis^[25]^. The Logit model is expressed as follows:

6 $ \mathrm{log}\left(\dfrac{P}{1-P}\right)={\beta }_{0}+{\beta }_{1}{X}_{1}+{\beta }_{2}{X}_{2}+\cdots +{\beta }_{n}{X}_{n} $

7 $ Odd\left(OR\right)={e}^{\beta } $

where *P* represents the probability of infestation, *β*_0_ is the model intercept, *β*_1_, *β*_2_, …, *β*_n_ are the regression coefficients for the independent variables *X*_1_, *X*_2_, …, *X*_*n*_.

The logistic regression model explains the relationship between independent and dependent variables through three key indicators. The regression coefficient (*β*) represents the direction (positive or negative), and strength of each variable's effect on infestation probability. The *p*-value tests the statistical significance of each variable, where a *p*-value less than 0.05 indicates that the variable is statistically significant in the model. The *Odds Ratio* (*OR*) quantifies the effect of changes in an independent variable on infestation probability, where *OR* > 1 indicates that an increase in the variable raises the probability of infestation, while *OR* < 1 indicates that an increase in the variable reduces the probability of infestation, with the calculation method given in Eq. (6). The greatest advantage of logistic regression is its ability to eliminate potential interaction effects or collinearity issues that may exist in one-way analysis, ensuring that the selected variables remain independently significant under multifactor conditions.

## Results

### Assessment of tree-level independence

Using Eq. (5), ICC[1] was calculated to be 0.19, indicating that the variation between plots has a relatively small impact on the probability of *P. armandii* infestation. The similarity among trees accounts for 19% of the total variance, with most of the variance explained by fixed effects, which is consistent with the assumption of independence.

### One-way comparative analysis

Firstly, among the growth characteristics of *P. armandii*, the DBH of the infested group was significantly larger than that of the non-infested group, with mean values of 16.94 and 15.00 cm, respectively (*p* < 0.01, see Table 1). This suggests that, with large DBH, *P. armandii* are more susceptible to infestation, indirectly reflecting that older trees are more prone to *D. armandi* attack. Similarly, tree height followed a comparable trend, with the infested group averaging 12.27 m, while the non-infested group averaged 11.53 m (*p* < 0.001), indicating that taller *P. armandii* may also have a higher risk of infestation. Openness and the minimum distance from *P. armandii* to the nearest non-*P. armandii* both had significant effects on the susceptibility of *P. armandii* to *D. armandi* infestation.

**Table 1:** Grouped statistics and significance test results.

Variable	Diseased_Mean ± std (SE)	Healthy_Mean ± std (SE)	*p*-value	Significance
DBH (cm)	16.94 ± 6.37 (0.17)	15.00 ± 5.46 (0.21)	< 0.001	Significant
Height (m)	12.27 ± 2.81 (0.08)	11.53 ± 3.69 (0.14)	< 0.001	Significant
Elevation	1752.50 ± 134.58 (3.61)	1699.28 ± 113.81 (4.44)	< 0.001	Significant
Slope	17.40 ± 5.82 (0.16)	18.99 ± 6.85 (0.27)	< 0.001	Significant
Slope aspect	100.01 ± 84.77 (2.27)	106.64 ± 88.89 (3.47)	0.110	Not significant
Openness, O	0.30 ± 0.18 (0.00)	0.27 ± 0.15 (0.01)	0.002	Significant
Nearest non-*Pinus armandii* distance	1.81 ± 1.40 (0.04)	1.67 ± 1.28 (0.05)	0.029	Significant
Uniform angle index, W	0.00: 7.33%	0.00: 8.07%	0.245	Not significant
	0.25: 17.11%	0.25: 14.61%		
	0.50: 44.57%	0.50: 47.95%		
	0.75: 19.34%	0.75: 16.95%		
	1.00: 11.65%	1.00: 12.79%		
Mingling, M	0.00: 12.51%	0.00: 7.91%	0.010	Significant
	0.25: 21.21%	0.25: 19.33%		
	0.50: 27.10%	0.50: 28.92%		
	0.75: 25.81%	0.75: 27.25%		
	1.00: 13.37%	1.00: 16.59%		
DBH dominance	0.00: 37.10%	0.00: 26.94%	< 0.001	Significant
	0.25: 28.97%	0.25: 28.92%		
	0.50: 18.76%	0.50: 24.66%		
	0.75: 9.78%	0.75: 14.00%		
	1.00: 5.39%	1.00: 5.48%		
Height dominance	0.00: 35.80%	0.00: 27.85%	< 0.001	Significant
	0.25: 29.26%	0.25: 28.01%		
	0.50: 19.19%	0.50: 23.19%		
	0.75: 10.14%	0.75: 14.00%		
	1.00: 5.61%	1.00: 6.85%		
For categorical variables, values are fixed at 0, 0.25, 0.5, 0.75, and 1, with respective proportions reported.				

Different levels of mingling and dominance significantly impact the probability of *P. armandii* being infested by *D. armandi*. Topographic conditions also play a significant role in the distribution of infested trees. The mean elevation of the infested group was 1,752.50 m, significantly higher than that of the non-infested group (1,699.28 m, *p* < 0.001, see Table 1), indicating that pest outbreaks are more frequent in higher-elevation areas. Similarly, the slope differed significantly between the two groups, with the mean slope in the infested group being 17.40°, lower than 18.99° in the non-infested group (*p* < 0.001, see Table 1), suggesting that areas with lower slopes are more prone to infestation.

**Figure 3:**
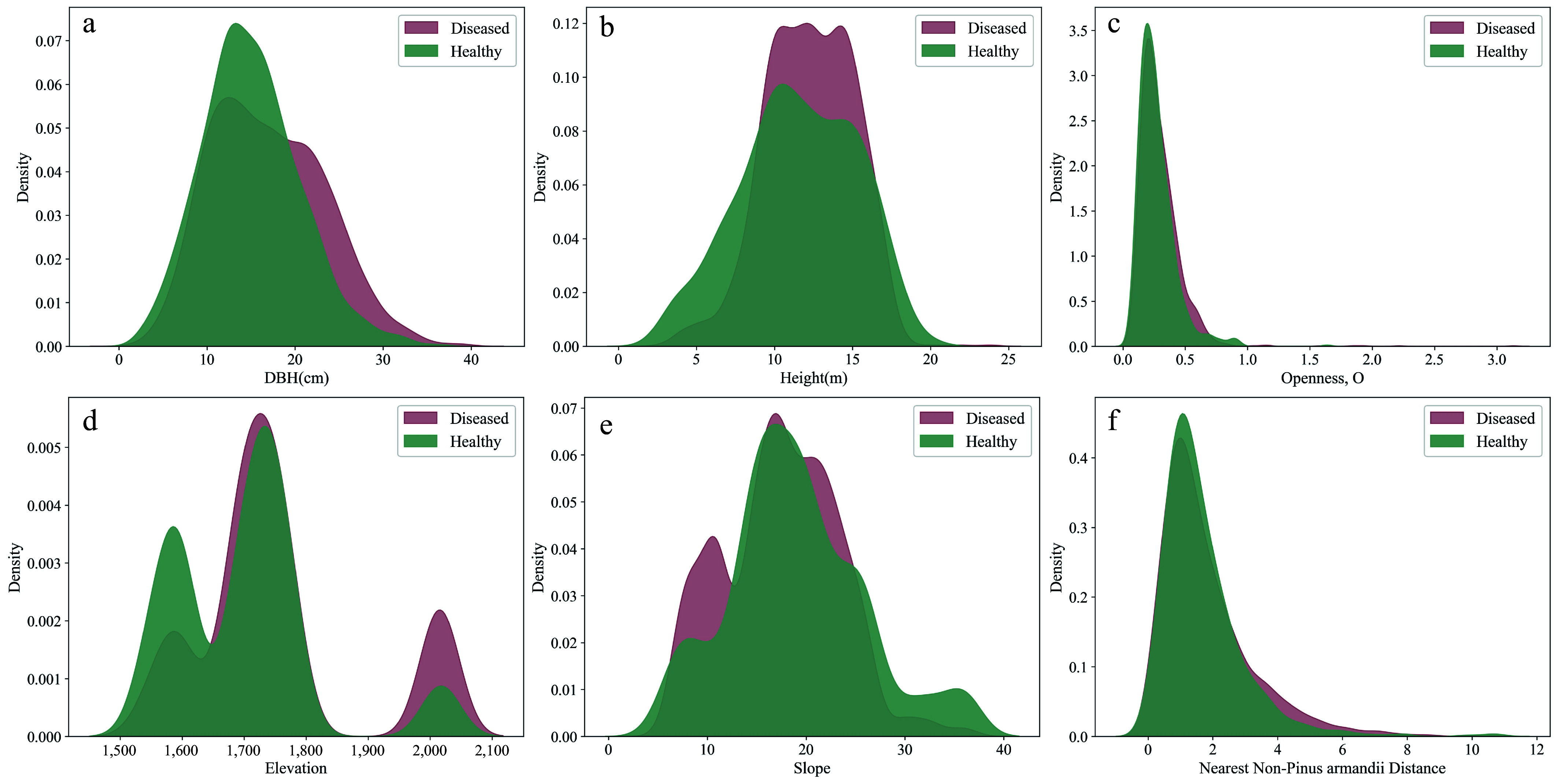
Distribution of continuous variables that showed significant effects on infestation probability between the infested and non-infested groups in univariate analysis.

**Figure 4:**
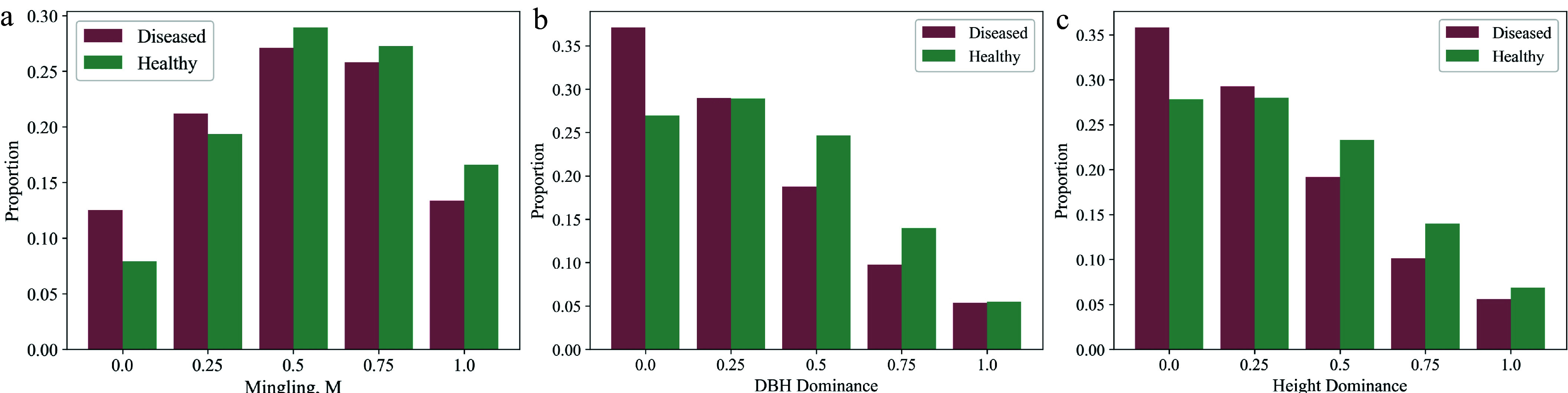
Proportional distribution of categorical variables that showed significant effects on infestation probability between the infested and non-infested groups in univariate analysis. The figure shows, for each category of every categorical variable, the proportion of infested trees among all infested trees, and the proportion of non-infested trees among all non-infested trees.

To visually represent the distribution characteristics of significant variables, kernel density estimation (KDE) plots and histograms were generated. The plots clearly illustrate that DBH and tree height distributions in the infested group skew to the right (Fig. 3a, b), meaning that trees with larger DBH and greater height are more susceptible to infestation. Additionally, the distributions of mingling, elevation, and slope show that the infested group is more concentrated in areas with higher mingling, higher elevation, and lower slope (Figs 3d & e, 4a). Furthermore, the distributions of DBH dominance and height dominance indicate that infested trees are more concentrated in areas where neighboring trees have lower DBH dominance and height dominance (Fig. 4b, c).

### Logit regression model analysis

To further explore the mechanisms by which different variables influence the risk of *P. armandii* infestation, a Logit regression model was constructed to evaluate the effects of mingling, DBH, tree height, elevation, dominance indices, and openness on infestation probability. The model results indicate that certain variables make significant contributions to predicting infestation probability (Table 2).

**Table 2:** Logit regression model results.

Variable	*β*	*p*-value	Odd	Significance
Constant	−5.205	< 0.001	0.005	Significant
DBH (cm)	0.044	0.003	1.045	Significant
Height (m)	0.023	0.422	1.023	Not significant
Openness, O	1.523	< 0.001	4.584	Significant
Nearest non-*Pinus armandii* distance	−0.085	0.138	0.919	Not significant
Elevation	0.003	< 0.001	1.003	Significant
Slope	−0.020	0.026	0.981	Significant
Mingling, M	−0.782	0.001	0.457	Significant
DBH dominance	−0.155	0.62	0.856	Not significant
Height dominance	0.133	0.666	1.143	Not significant

Among tree growth characteristics, DBH had a significant positive effect on the probability of *D. armandi* infestation (*p* = 0.003), with an *OR* of 1.045. This means that for every 1 cm increase in DBH, the probability of infestation increases by approximately 4.5%. In contrast, tree height was not a significant factor (*p* = 0.422), suggesting that tree height may not be a key variable influencing infestation risk. Regarding stand structure, mingling had a significant negative effect on infestation (*p* = 0.001), with an *OR* of 0.457, indicating that for each unit increase in mingling, the risk of infestation decreases by approximately 54.3%. This highlights the important role of species mingling in reducing *P. armandii* susceptibility to *D. armandi* infestations. Similarly, openness had a significant positive effect on infestation probability (*p* < 0.001), with an *OR* of 4.584, meaning that for every unit increase in openness, the infestation risk increases by approximately 358.4%. This suggests that higher openness environments facilitate *D. armandi* infestation. Regarding topographic conditions, elevation had a significant positive effect on infestation risk (*p* < 0.001), with an *OR* of 1.003, indicating that for every 1 m increase in elevation, the probability of infestation increases by 0.3%. Slope showed a significant negative effect on infestation probability (*p* = 0.026), with an *OR* of 0.981, suggesting that for every 1° increase in slope, the probability of infestation decreases by approximately 1.9%.

### Influence of significant variables on infection probability

**Figure 5:**
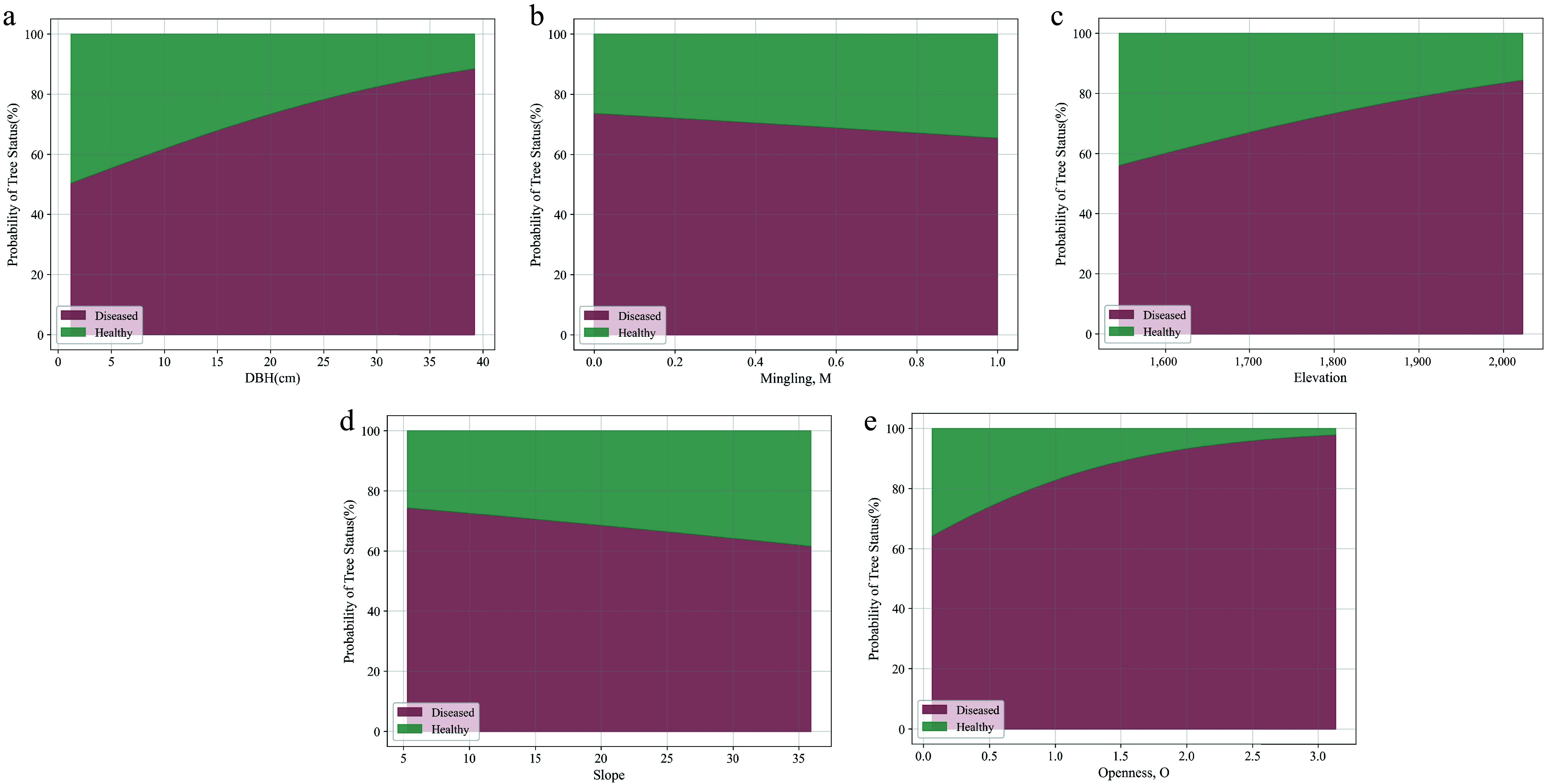
Predicted probability distribution of trees in the 'Healthy' or 'Diseased' state under different variable conditions, with the total probability across all variables summing to 100%.

This study employed the logistic regression model to analyze the influence of significant variables (mingling, DBH, elevation, slope, and openness) on the probability of infection, further revealing the mechanisms by which these variables affect infection risk. Figure 5, as a probability contour plot, clearly illustrates the relative probabilities of infestation and health at different levels of variable changes^[26]^. The results indicate that different significant variables exhibit notable differences in their impact on infection probability across their respective ranges. Figure 5 presents the predicted probability distributions of trees being in either the 'Healthy' or 'Diseased' state under different variable conditions, with the total probability across all variables summing to 100% at any given point.

As shown in Fig. 5a, as mingling gradually increases from 0 to 1, the infection probability significantly decreases, initially dropping from approximately 73.64% to 65.39%. This suggests that higher mingling (i.e., greater species diversity) can effectively reduce the risk of insect infestation. Figure 5b illustrates that DBH has a significant positive correlation with infection probability. As DBH increases from 5 to 40 cm, the infection probability rises steadily from approximately 50.41% to 88.44%. This trend indicates that trees with larger DBH have a higher risk of infection. This may be because the phloem of larger trees provides a more suitable host environment for insects, resulting in a greater risk of infestation. Elevation also exhibits a significant positive impact on infection probability (Fig. 5c). As elevation rises from 1,500 to 2,100 m, the infection probability increases from approximately 56.09% to 84.38%. Figure 5d demonstrates the negative effect of slope on infection probability. As the slope increases from 5° to 35°, the infection probability significantly decreases, dropping from 74.34% to 61.57%. This suggests that steeper slopes may hinder the spread of insects. Figure 5e illustrates the positive effect of Openness on the probability of infestation. As openness increases from 0 to 3, the probability of infestation rises significantly, from 64.25% to 97.85%.

### Statistical analysis of ecological suitability of neighboring tree species

**Figure 6:**
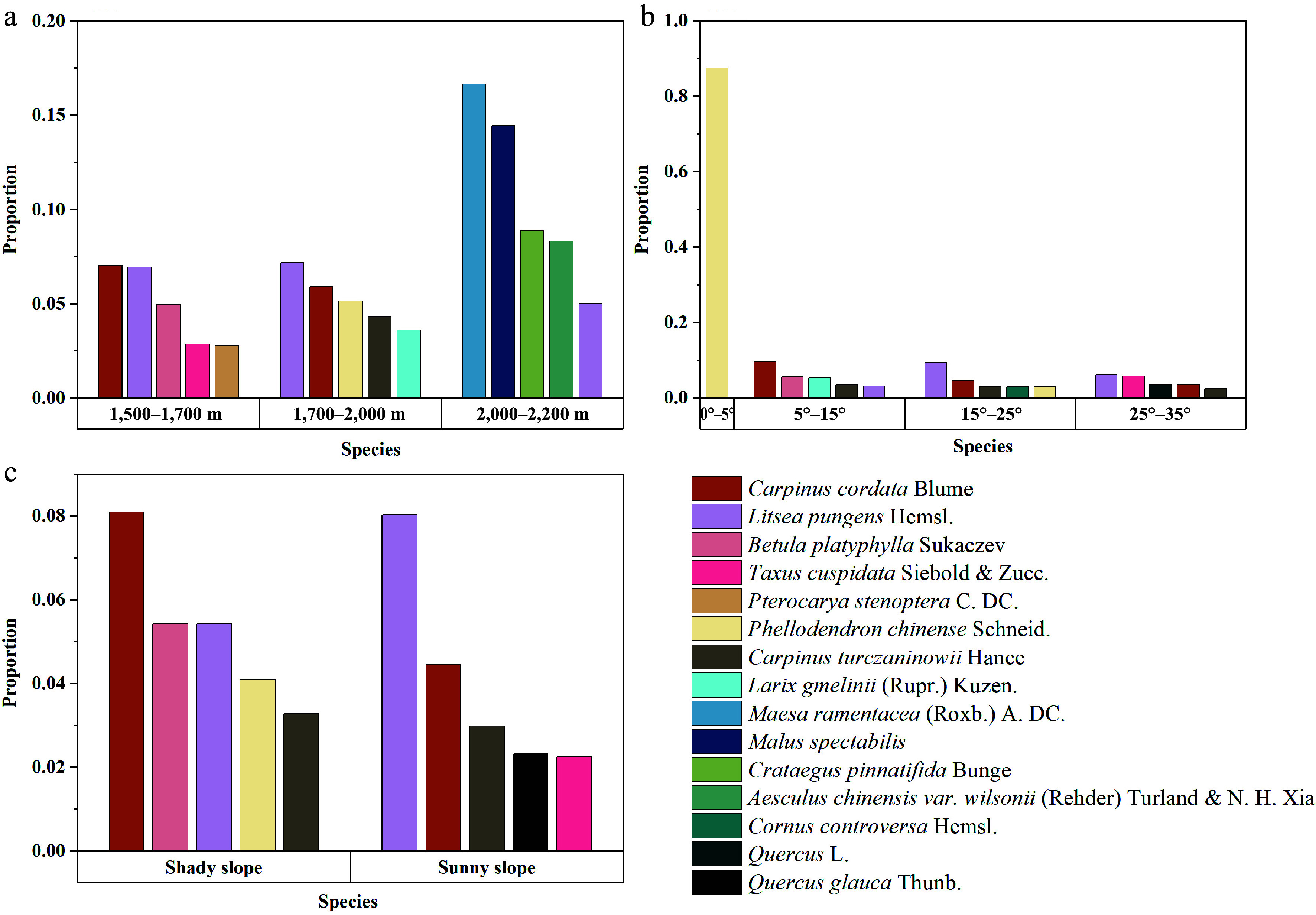
Statistical data for the nearest neighbors of *P. armandii* trees, grouped by different elevation levels, slope aspects, and slope gradient categories. The species proportions within each independent terrain classification interval are sorted in descending order of tree species count, though a higher proportion does not necessarily represent a higher number of trees. (a) The top 5 neighboring trees with the highest proportions in different elevation ranges. (b) The top 5 neighboring trees with the highest proportions in different slope gradient ranges. (c) The top 5 neighboring trees with the highest proportions under different slope aspect conditions.

A total of 657 healthy *P. armandii* trees were analyzed along with their four neighboring trees. The five most abundant neighboring species in different terrain conditions were identified, and the results are shown in Fig. 6. The data were grouped according to different elevation levels, slope aspects, and slope gradients, and the tree species were ranked according to the proportion of species within independent terrain intervals, from largest to smallest. However, a larger proportion does not necessarily indicate a higher number. The elevation was divided into three groups: 1,500−1,700 m, 1,700−2,000 m, and 2,000−2,200 m; the slope was divided into four levels: 0°−5°, 5°−15°, 15°−25°, and 25°−35°; the slope aspect was categorized into sunny slope and shady slope.

Based on the statistical results, the distribution of neighboring tree species around *P. armandii* shows clear variation across different elevation intervals. A total of 12 tree species were recorded in the three elevation intervals. *Carpinus cordata* Blume and *Litsea pungens* Hemsl. had higher proportions in the 1,500−2,000 m range. *L. pungens* also had a small distribution in the 2,000−2,200 m range, indicating a strong environmental adaptability. In comparison to these two species, other tree species have narrower ecological niches and are more abundant in specific elevation ranges. For example, *Betula platyphylla* Sukaczev and *Taxus cuspidata* Siebold & Zucc. had proportions of 4.98% and 2.86%, respectively, in the 1,500−1,700 m range. *Phellodendron chinense* Schneid. and *Carpinus turczaninowii* Hance had proportions of 5.15% and 4.33%, respectively, in the 1,700−2,000 m range. *Maesa ramentacea* (Roxb.) A. DC. and *Malus* spectabilis had proportions of 16.67% and 14.44%, respectively, in the 2,000−2,200 m range.

Regarding slope, since the Shennongjia forest area is predominantly mountainous with limited flat or low-slope land, the sample size for the 0°−5° range was small. The neighboring species *P. chinense* accounted for as much as 87.50%, with other species being *P. armandii*. In the 5°−35° range, both *C. cordata* and *L. pungens* had certain distributions. *C. cordata* is more suitable for slopes in the 5°–15° range, while *L. pungens* is better adapted to slopes between 15° and 35°. *B. platyphylla* and *T. cuspidata* had some distributions in both low and high slope ranges, with proportions of 5.60% and 5.88%, respectively. Additionally, *C. turczaninowii* showed good adaptability to slope changes, with distributions across the 5°−35° slope range.

In terms of slope aspect, *C. cordata* had the largest proportion in the shady slope, accounting for 8.10%, while *L. pungens* and *B. platyphylla* each accounted for 5.43%, and *P. chinense* had a relatively high proportion of 4.09%. In contrast, on sunny slopes, *L. pungens* had the highest proportion at 8.05%, while *C. cordata* accounted for 4.46%, still higher than the other three species. *C. turczaninowii* showed similar proportions on both shady and sunny slopes, indicating its strong adaptability to slope aspect.

By combining the species distribution in different terrain intervals, it can be concluded that *L. pungens* is suitable for growing at elevations between 1,500 and 2,000 m, with slopes between 15° and 35°. Slope aspect has little effect on its growth, but it thrives better on sunny slopes. *C. cordata* is best suited for growing at elevations between 1,500 and 2,000 m, with slopes between 5° and 35°, preferably on shady slopes. *P. chinense* is predominantly found in the 1,700−2,000 m range, with slopes less than 5°, and on shady slopes. *B. platyphylla* has stronger environmental preferences and is mainly found in the 1,500−1,700 m range, with slopes between 5° and 15° on shady slopes. As for the 2,000−2,200 m range, due to the higher altitude and harsher growing conditions, fewer tree species are found. While the remaining four species, except for *L. pungens*, have higher distribution percentages, their distributions across other terrain factors were not as high. Therefore, they are not considered suitable as primary species for mixing with *P. armandii*. In contrast, *L. pungens* can adapt to environments above 2,000 m and shows strong adaptability to other terrain changes, making it a preferred candidate for mixed planting with *P. armandii* in conditions with high elevation (2,000−2,200 m) and steep slopes.

## Discussion

### Impact of terrain on the resistance of *P. armandii* to pest infestation

The results of this study indicate that with an increase in elevation, the probability of *P. armandii* being affected by pests significantly increases, which is consistent with findings from other related studies^[4]^. Elevation affects ecological factors such as temperature, humidity, and precipitation to a certain extent. High-altitude areas typically have lower temperatures, higher humidity, and relatively simple vegetation composition, which may lead to more concentrated host resources for *D. armandi*, increasing the risk of pest outbreaks^[27]^. Compared to low-altitude areas, the richness of natural enemies or competitors may be lower at high altitudes, weakening the natural control capacity for *D. armandi* populations. This result is also in line with the characteristics of high-density *P. armandii* forests and simple stand structure at high altitudes in the Shennongjia forest area^[7,27]^.

Furthermore, the significant result for slope indicates that the steeper the slope, the lower the risk of *P. armandii* infestation, which is consistent with other studies on *D. armandi*^[4]^. This may be related to differences in the distribution of light, heat, water resources, and host density on steep slopes. Steep areas experience faster water runoff, which may lead to lower soil moisture, a condition unfavorable for the reproduction and spread of *D. armandi*^[7]^. In addition, the density of *P. armandii* in steep areas may be lower, further reducing the potential for pest transmission among host trees.

In this study, slope aspect did not show a significant effect on the probability of infestation. Although slope aspect can influence ecosystems by modulating light and thermal conditions, its effect in the Shennongjia study area may have been overshadowed by other significant variables. Furthermore, the climate in the Shennongjia forest area is relatively balanced, and the microclimatic effects of slope aspect may not have shown a significant role in the pest infestation process of *D. armandi*^[28]^.

### Impact of stand structure on the resistance of *P. armandii* to pest infestation

The results of this study show that with increasing mingling, the probability of *P. armandii* infestation significantly decreases. Mixed forests generally have stronger ecological stability and resistance to pests compared to pure forests, which is consistent with the findings of existing studies^[10,13]^. When mingling reaches 1, meaning that the four nearest neighboring trees around *P. armandii* are all non-host species, the probability of infestation significantly drops. This may be related to the volatile substances emitted by non-host plants. Previous studies have shown that volatile organic compounds from plants can have a significant impact on herbivorous insects' host selection behavior^[2]^. The substances emitted by non-host plants may have a repellent effect, interfering with *D. armandi*'s ability to locate host plants. Additionally, increasing mingling not only alters the chemical signaling environment but may also reduce pest outbreaks by changing the spatial isolation of hosts^[29]^. When *P. armandii* is surrounded by non-host plants, the efficiency of *D. armandi* moving between hosts may decrease, thus inhibiting the spread of pest damage.

Although many studies have pointed out that mixed forests are more resistant to pests than pure forests, other research indicates that the specific arrangement of tree species may be more important than simple mingling^[30]^. For example, in some cases, if the proportion of non-host plants is too high, it may provide a suitable habitat for pests, weakening their repellent effects. Therefore, although this study confirmed the positive impact of mingling on the resistance of *P. armandii* to pests, further research is needed to explore the role of non-host species types, distribution patterns, and their spatial relationship with host plants in pest control.

The results of this study indicate that *P. armandii* individuals with larger DBH are more susceptible to *D. armandi* infestation, and the probability of infestation significantly increases with larger DBH. This suggests that DBH, an important indicator of tree growth, is closely related to *P. armandii*'s ability to resist pest damage. Insects typically exhibit clear preferences in host selection, and *D. armandi* tends to infest trees with larger DBH, which is consistent with its biological characteristics and ecological needs. On one hand, *P. armandii* trees with larger DBH usually represent older, more mature individuals with thicker phloem, providing more abundant nutrients and better palatability, which benefits *D. armandi* feeding and reproduction^[31]^. Therefore, reasonable forest management strategies can be developed, such as harvesting *P. armandii* trees with larger DBH that are approaching maturity.

The results of this study indicate that openness has a significant impact on the resistance of *P. armandii* to pest infestation, with higher openness leading to a higher probability of *P. armandii* being infested by *D. armandi*. This finding contradicts many related studies, which generally suggest that higher openness helps reduce pest occurrence, as higher light intensity and lower humidity may limit the survival and reproduction of pests^[32]^. However, in this study, forests with higher openness exhibited higher pest infestation risks. Several factors may explain this. First, increased openness may lead to changes in light conditions, which directly affect the growth and health of *P. armandii*. Higher light intensity and greater daily light variation may result in increased water evaporation from the trees, thereby affecting their physiological health and making them more susceptible to *D. armandi*. Additionally, in forests with larger openness, although air circulation is enhanced, pests may also spread more easily within the forest. Compared to more closed forests, pests can spread over greater distances in open spaces, leading to quicker infestation of multiple trees^[33]^.

### Management strategies for *P. armandii* forests

To effectively improve the pest resistance of *P. armandii* forests and reduce the risk of *D. armandi* infestation, a comprehensive forest management strategy must be implemented, covering aspects such as harvesting, thinning, and forest structure adjustment.

First, regarding harvesting strategies, a reasonable harvesting plan should be developed for *P. armandii* forests approaching maturity. Regular harvesting and replacing older trees helps reduce overcrowding and aging, which lowers pest transmission pathways and prevents the accumulation of pests and diseases in the forest. In high-altitude areas and regions with low slopes, targeted harvesting of *P. armandii* trees with higher infestation probabilities should be carried out to prevent these areas from becoming sources of infestation. Through planned harvesting, forest health can be improved, and the risk of pest spread can be effectively reduced^[34]^.

Thinning strategies are also critical. In forest management, care should be taken to avoid creating overly open canopy spaces, as this helps reduce the environment's suitability for *D. armandi* reproduction, which thrives in certain light and humidity conditions. By increasing spacing between standing trees, limiting openness, and promoting the development of multi-aged forests, the forest's site conditions and structural diversity can be improved. This, in turn, increases resistance to *D. armandi* movement, preventing pest spread^[35]^.

Regarding mixed forest conversion, higher mingling effectively reduces the proportion of infested trees and enhances forest resilience^[36]^. Therefore, gradually converting pure *P. armandii* forests into mixed forests is a key measure for improving pest resistance. The selection of mixed species should be optimized based on local tree species and topographic factors such as elevation and slope^[37]^. In the Shennongjia region, it is recommended to choose adaptive native species such as *L. pungens*, *C. cordata*, and *B. platyphylla* to mix with *P. armandii* in areas at elevations of 1,500 to 1,700 m with steeper slopes. For areas at elevations of 1,700 to 2,000 m, *C. turczaninowii* is recommended as a mixed species, while *P. chinense* is suitable for mixing with healthy *P. armandii* on flat terrain to reduce the risk of infestation. For the 2,000 to 2,200 m elevation range, due to the special topographic conditions, only *L. pungens* has good adaptability and can be considered as the priority mixed species.

### Observational data

Although this study found strong correlations between several factors and the probability of *P. armandii* infestation, it is important to note that correlation does not imply causation. There may be confounding factors that influence both the correlated variables simultaneously, making them appear related. For example, studies have shown a significant correlation between tree species diversity and the risk of tree infestation by pests^[38]^. However, there is also evidence that climate conditions and soil properties may influence both tree species diversity and pest infestation mechanisms, thus playing an important role in the relationship between the two^[39]^.

### Random effect of plot

It is generally considered in both domestic and international research that a low ICC[1] value indicates that, after accounting for fixed effects, the contribution of random effects to the model is relatively small^[23,40]^. In this study, the calculated ICC[1] = 0.19 reflects the degree to which plot-level differences affect tree mortality. This relatively low value is usually interpreted as indicating that differences between plots are not significant in the model of tree mortality, though standards may vary across different fields and research contexts. There is no absolute unified standard in forestry and ecology. Even when the ICC[1] value is high, it does not necessarily mean that random effects have an absolute impact on tree infestation risk within plots, as fixed effects may explain most of the variability^[41]^.

A central aim of this study was to investigate whether environmental variations within the same classification levels (e.g., elevation, slope) similarly affect the probability of *P. armandii* infestation. These fixed effects may significantly absorb the variance otherwise attributed to random effects, or the random effects themselves may originate from these fixed factors. For example, variation in elevation across plots may drive differences in microclimate and soil properties. Therefore, fixed effects in this study have largely weakened the contribution of random effects. Forcing the inclusion of plot ID as a random effect in the model may impair the interpretability of these fixed effects^[42,43]^.

Therefore, the ICC[1] value in this study indicates that the contribution of random effects to the model is small, and the random effect of plots can be considered negligible to some extent. However, some unaccounted fixed effects still exist, which may influence the completeness of the model and the explanation of plot-level differences. The interpretation of plot differences still relies on further in-depth exploration and refinement of fixed effects in future research. Further studies could introduce more environmental and management factors to verify this conclusion and improve the model's accuracy.

## Conclusions

This study conducted field investigations and statistical analyses at the individual tree scale in the Shennongjia forest area to explore the effects of topographic features, horizontal structure characteristics, and individual tree traits on the probability of *P. armandii* being infected by *D. armandi*. First, elevation and slope had significant impacts on the probability of *P. armandii* being infested. Higher elevation and lower slope increased the likelihood of *P. armandii* being infected by *D. armandi*. Additionally, the study confirmed that mingling significantly influenced the infection probability: higher mingling reduced the risk of *P. armandii* infestation. Finally, DBH was identified as one of the key drivers affecting infection probability, with larger *P. armandii* trees exhibiting a significantly higher risk of infestation.

Based on the analysis results, several forest transformation and species configuration strategies are proposed to reduce the infection risk of *P. armandii*. To minimize infestation risk, afforestation of *P. armandii* should prioritize areas below 2,000 m elevation and with steeper slopes. In cases where environmental conditions cannot be altered, increasing mingling is also an effective measure to lower the risk of infestation.

For tree species configuration, reducing openness as much as possible while increasing mingling can enhance the pest resistance of *P. armandii*. Native species such as *L. pungens*, *C. cordata*, and *B. platyphylla* are well-suited for steep-slope sites between 1,500 and 1,700 m elevation and should be prioritized as companion species for mixed planting with *P. armandii*. *P. chinense* is suitable for planting in flat areas at elevations between 1,700 and 2,000 m alongside healthy *P. armandii* to reduce infestation risk. Lastly, in regions between 2,000 and 2,200 m elevation, *L. pungens* should be the primary choice for mixed planting when transforming *P. armandii* forests.

## Data Availability

The datasets generated and/or analyzed during the current study are not publicly available to protect sensitive geographical information of forest plots and detailed growth status of trees within the plots, but they are available from the corresponding author upon reasonable request.
